# The effect of attention on wellbeing in combat sport athletes: the mediating role of competitive anxiety

**DOI:** 10.3389/fpsyg.2026.1870752

**Published:** 2026-07-13

**Authors:** Sermet Toktaş, Faik Öz, Berkay Ayverdi, Çetin Tan, Suriye Tan, Selin Biçer Baikoğlu, İrfan Kara, Sermin Ağralı Ermiş, Gamze Akyol, Kadir Tiryaki, Elif Taşkuyu, Fatma Neşe Şahin

**Affiliations:** 1 Department of Physical Education and Sports Teaching, Faculty of Sport Sciences, Adıyaman, Türkiye; 2 Department of Exercise and Sport Sciences, Faculty of Sport Sciences, Avrasya University, Trabzon, Türkiye; 3 Ankara University Faculty of Sport Sciences, Ankara, Türkiye; 4 Division of Psychosocial Areas in Sport, Department of Physical Education and Sports Teaching, Faculty of Sport Sciences, Fırat University, Elazığ, Türkiye; 5 Quality Management Unit, Fırat University Hospital, Elazığ, Türkiye; 6 Division of Sport Health Sciences, Department of Coaching Education, Faculty of Sport Sciences, Istanbul University-Cerrahpaşa, Istanbul, Türkiye; 7 Department of Sport Management, Faculty of Sport Sciences, Istanbul Gelişim University, Istanbul, Türkiye; 8 Faculty of Sport Sciences, Aydın Adnan Menderes University, Aydın, Türkiye; 9 Department of Physical Education and Sport, Faculty of Sport Sciences, Düzce University, Düzce, Türkiye; 10 Faculty of Sport Sciences, Akdeniz University, Antalya, Türkiye; 11 Division of Sport Health Sciences, Department of Coaching Education, Ankara University Faculty of Sport Sciences, Ankara, Türkiye

**Keywords:** attentional control, emotion regulation, mediation model, performance psychology, psychological regulation

## Abstract

**Introduction:**

This study aimed to examine whether the effect of attentional processes on psychological well-being in combat sport athletes operates directly or indirectly through competitive anxiety within an integrated model.

**Methods:**

A total of 418 actively competing athletes participated in the study, and the relationships among athlete attention, competitive anxiety, and psychological well-being were analyzed using a regression-based mediation model.

**Results:**

The findings revealed that athlete attention had a strong and positive effect on psychological well-being (β = 0.718), while also showing a significant negative effect on competitive anxiety (β = 0.574). In turn, competitive anxiety was found to negatively predict psychological well-being (β = −0.242). When competitive anxiety was included in the model, the direct effect of attention on psychological well-being decreased (β = 0.579), indicating the presence of a mediation structure. Bootstrap analysis further confirmed that the indirect effect was statistically significant (β = 0.139, 95% CI [0.091, 0.202]), demonstrating that competitive anxiety plays a partial mediating role in this relationship.

**Discussion:**

These results suggest that attentional processes are associated with psychological well-being both directly and indirectly through their statistical association with competitive anxiety. From a theoretical perspective, the findings support the assumptions of Attentional Control Theory within a sport context and highlight the role of attention as a multidimensional regulatory mechanism. Practically, the findings suggest that interventions targeting both attentional control and anxiety regulation may be associated with more favorable psychological well-being outcomes than approaches focusing solely on performance-related outcomes.

## 1 Introduction

Although the detrimental effects of competitive anxiety have long been consistently documented in the athletic performance literature, the specific cognitive processes through which this effect operates have not yet been fully clarified. Contemporary theoretical approaches suggest that anxiety does not impair performance directly; rather, it exerts its influence indirectly by disrupting cognitive control systems (Mellalieu et al., 2006; Englert and Bertrams, 2012). Attentional control refers to an individual’s capacity to direct attention to task-relevant stimuli, maintain that focus, and inhibit distracting information (Burgoyne and Engle, 2020). Increased anxiety leads to a shift of attention toward threat-related stimuli and a reduction in the efficient allocation of cognitive resources. In explaining this relationship, Attentional Control Theory, which emphasizes the impact of anxiety on cognitive control systems, provides a key theoretical foundation (Eysenck et al., 2007). This perspective indicates that attentional processes are not limited to performance outcomes but are also associated with broader psychological functioning (Eysenck et al., 2007; Bar-Haim et al., 2007; Moriya and Sugiura, 2012). Recent findings indicate that attentional control is associated not only with performance but also with emotion regulation and psychological wellbeing. The literature explaining the relationship between attentional processes and psychological wellbeing generally converges around two main perspectives. One line of research suggests that attentional control is positively associated with psychological wellbeing and functions as a cognitive resource that supports the regulation of emotional processes (Nielsen et al., 2019; Chu et al., 2021; Koç et al., 2025).

The alternative perspective posits that attentional control influences psychological outcomes indirectly by regulating anxiety levels, with this effect operating through emotion regulation processes (Eysenck et al., 2007; MacDonald and Olsen, 2020). However, comprehensive approaches that simultaneously test these two explanatory pathways within a single model remain limited. At this point, a central question emerges: does the effect of attentional processes on psychological wellbeing occur directly, or does it arise indirectly through anxiety regulation mechanisms? This relationship becomes more pronounced in sport settings, where cognitive and emotional demands are particularly intense.

In combat sports, competitive anxiety is conceptualized as a multidimensional construct that affects not only performance but also athletes’ psychological adjustment and wellbeing. Due to heightened threat perception, direct opponent interaction, and the need for rapid decision-making, combat sports provide a performance context in which attentional and anxiety processes are experienced more intensely. It has been reported that psychological resilience and related individual characteristics are significantly associated with lower levels of cognitive and somatic anxiety, and that systematic links exist between anxiety and psychological wellbeing (Mojtahedi et al., 2023; Derelioğlu et al., 2025; Akıllıoglu and Cherkashin, 2026). Interventions targeting attentional processes, particularly those based on attentional control and mindfulness approaches, have been shown to enhance emotion regulation capacity and reduce anxiety levels (Rogowska and Tataruch, 2024; Zhong et al., 2025). Taken together, these two lines of research support the theoretical view that the effect of attentional processes on psychological wellbeing is better explained by an indirect mechanism operating through anxiety regulation, rather than by a direct cognitive advantage. However, the existing literature has largely examined attentional control, competitive anxiety, and psychological wellbeing as independent constructs, and studies that investigate these variables jointly within a single model and test their relationships through a mediational mechanism remain limited (Shi et al., 2024; Johles et al., 2025). This gap necessitates testing whether the effect of attentional processes on psychological wellbeing occurs directly or indirectly via anxiety regulation within a comprehensive model framework. Accordingly, the present study aims to examine the relationships among attentional control, competitive anxiety, and psychological wellbeing within an integrated model, and to elucidate the underlying mechanisms linking these variables.

### 1.1 Theoretical framework

Consistent with Attentional Control Theory (Eysenck et al., 2007), attentional processes represent a core component of cognitive control, enabling individuals to selectively allocate attention to goal-relevant stimuli while inhibiting distracting information. Beyond their role in performance, attentional processes have been increasingly linked to broader psychological functioning, particularly psychological wellbeing. Individuals with higher levels of attentional control tend to exhibit greater emotional stability, more effective self-regulation, and lower psychological distress (MacDonald and Olsen, 2020). Empirical evidence further suggests that attention-focused interventions enhance positive affect and overall wellbeing (Tang et al., 2019), while attentional biases toward positive stimuli are associated with higher life satisfaction and wellbeing (Xu et al., 2015). Within this framework, attentional processes can be understood as a cognitive resource that directly contributes to psychological wellbeing by supporting adaptive emotional and cognitive functioning.

Attentional control plays a decisive role in regulating both performance and emotional responses through an individual’s capacity to allocate and sustain cognitive resources. As anxiety levels increase, the individual’s ability to consciously direct attention is weakened, leading to a loss of control over attentional focus and a shift toward environmental stimuli (Eysenck et al., 2007). This shift results in a tendency to orient attention toward threat-related cues and reduces the efficient use of cognitive resources. These effects become more pronounced in sport settings, where elevated anxiety undermines attentional control and negatively influences performance-related cognitive processes (Wilson, 2008). However, individuals with stronger attentional control have been observed to be more resilient to these disruptive effects and to exhibit lower levels of anxiety during competition (Tomé-Lourido et al., 2019). In addition, attention-based interventions have been shown to be effective in reducing anxiety levels and in promoting a more balanced psychological state among athletes (Wang et al., 2024). Taken together, these findings indicate that attentional processes function as a key factor in regulating competitive anxiety, and that higher levels of attentional control are associated with lower anxiety levels.

Competitive anxiety is considered a fundamental psychological factor that influences individuals’ cognitive and emotional functioning in sport settings. As anxiety levels increase, the individual’s capacity to sustain and direct attention is weakened, leading to a shift of focus toward uncontrollable stimuli and adversely affecting emotion regulation processes. This disruption in emotional balance results in impairments in subjective evaluations, which are associated with lower levels of psychological wellbeing. Empirical studies on athletes have demonstrated that increases in competitive anxiety are linked to decreases in subjective happiness and psychological wellbeing (Zhang et al., 2024; Özkan et al., 2026). Moreover, higher levels of anxiety have been reported to be associated with burnout and diminished psychological functioning (Yang et al., 2024). Meta-analytic findings indicating that interventions targeting competitive anxiety can effectively reduce anxiety levels further support the role of this construct in shaping psychological outcomes (Ong and Chua, 2021). Taken together, these findings suggest that competitive anxiety is negatively associated with psychological wellbeing.

Explaining the relationship between attentional processes and psychological wellbeing solely in terms of a direct cognitive advantage appears insufficient. Attentional control influences which stimuli individuals focus on, thereby affecting the intensity and duration of anxiety experiences. As anxiety levels increase, the capacity to sustain attention weakens, leading to a shift toward threat-related stimuli and adversely affecting emotion regulation processes (Eysenck et al., 2007). Disruptions in emotional balance are, in turn, associated with lower levels of psychological wellbeing. Empirical studies on athletes have shown that competitive anxiety is linked to negative psychological outcomes such as burnout (Yang et al., 2024). Moreover, meta-analytic findings demonstrating that interventions targeting competitive anxiety can effectively reduce anxiety levels further support the role of this variable in shaping psychological outcomes (Ong and Chua, 2021). Taken together, these findings suggest that the effect of attentional processes on psychological wellbeing is not purely direct but is also indirectly shaped through changes in competitive anxiety, with anxiety potentially serving a partial mediating role in this relationship. Although alternative model specifications may be theoretically plausible, the present study was guided by Attentional Control Theory (Eysenck et al., 2007), which conceptualizes attentional control as a higher-order cognitive process associated with the regulation of anxiety-related responses. Accordingly, competitive anxiety was examined as a potential statistical pathway linking attentional processes to psychological wellbeing. Future studies may explore alternative model configurations among these variables.

### 1.2 Hypotheses

The hypotheses developed to be tested within the scope of this study are as follows:

**H1:** Higher levels of attentional processes are associated with higher levels of psychological wellbeing among combat sport athletes.

**H2:** Attentional processes have a significant negative effect on competitive anxiety.

**H3:** Competitive anxiety has a significant negative effect on psychological wellbeing.

**H4:** Competitive anxiety mediates the relationship between attentional processes and psychological wellbeing.

## 2 Materials and methods

### 2.1 Research design

In this study, a cross-sectional quantitative research design was employed to examine the relationships among attentional processes, competitive anxiety, and psychological wellbeing in combat sport athletes. The study was structured within a regression-based mediation model framework, allowing for the testing of both direct and indirect effects among the variables (Creswell and Creswell, 2018). Data collection was conducted following ethical approval, and the data were gathered between August 2025 and November 2025. Data were collected during athletes’ regular training and sport participation periods rather than immediately before a specific competition. Consequently, the scores obtained from the Competitive State Anxiety Inventory-2 (CSAI-2) should be interpreted as reflecting athletes’ perceived competitive anxiety within their broader sport context rather than anxiety assessed immediately prior to a particular competition. Inclusion criteria were defined as follows: being 18 years of age or older; being an actively licensed athlete in any combat sport discipline; engaging in regular training; having at least 1 year of sport experience; and providing voluntary consent to participate in the study. Exclusion criteria were defined as follows: being under 18 years of age; self-reporting a psychiatric diagnosis or ongoing psychological treatment; providing incomplete or erroneous responses to the data collection instruments; and exhibiting outliers or inconsistent response patterns identified during the data analysis process.

**FIGURE 1:**
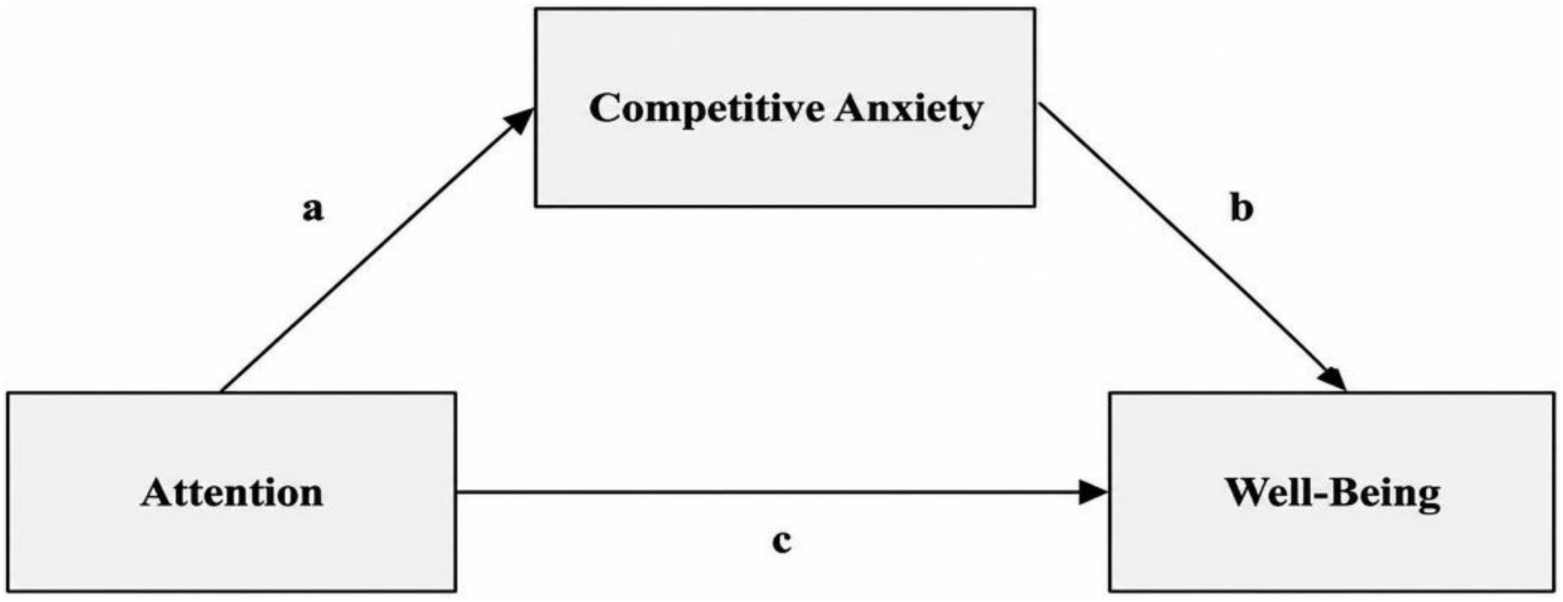
The research model. Path a represents the effect of athlete attention on competitive anxiety; Path b represents the effect of competitive anxiety on psychological well-being; Path c’ represents the direct effect of athlete attention on psychological well-being. The indirect effect is represented by the product of paths a and b (a × b), whereas path c represents the total effect of athlete attention on psychological well-being.

The research model developed within the scope of the study is presented in Figure 1. In this model, the effect of attentional processes on psychological wellbeing was examined through the mediating role of competitive anxiety. Accordingly, the effect of attention on competitive anxiety is represented as path “a,” the effect of competitive anxiety on psychological wellbeing as path “b,” and the direct effect of attention on psychological wellbeing as path “c’.” The indirect effect was calculated as the product of paths a and b (a × b), while the total effect was evaluated as the sum of the direct and indirect effects.

### 2.2 Participants

The participant group of the study consisted of a total of 418 athletes who were actively engaged in training and competition across various combat sport disciplines. A purposive sampling approach was employed to recruit actively competing combat sport athletes who met the predefined inclusion criteria. Participants were recruited through combat sport clubs and coaches during the data collection period. The sampling strategy was based on the intentional selection of individuals who were actively engaged in combat sports and were considered suitable for addressing the objectives of the study. Accordingly, individuals who were actively licensed athletes in combat sports, where the psychological demands of competition are particularly pronounced, were targeted for inclusion. Participants were informed about the purpose of the study and the voluntary nature of their participation and were evaluated based on the following inclusion criteria: being over 18 years of age, not having a diagnosed psychological disorder, and being actively involved in combat sports. Individuals under the age of 18, those with a diagnosed psychological condition, or those not actively competing were excluded from the study.

Demographic characteristics of the participants are presented in Table 1.

**TABLE 1:** Demographic characteristics of the participants.

Variable	Category	f	%
Gender	Male	197	47.1
	Female	221	52.9
Age	18–22 years	169	40.4
	23–27 years	125	29.9
	28–32 years	86	20.6
	33 years and above	38	9.1
Years of sport experience	1–5 years	161	38.5
	6–10 years	128	30.6
	11–15 years	90	21.5
	16 years and above	39	9.3
Athletic level	Amateur	331	79.2
	Professional	87	20.8
National athlete status	Yes	149	35.6
	No	269	64.4
	Total	418	100.0

Table 1 presents the demographic characteristics of the 418 athletes who participated in the study. Of the participants, 52.9% were female and 47.1% were male. Regarding age distribution, 40.4% were between 18 and 22 years, 29.9% between 23 and 27 years, 20.6% between 28 and 32 years, and 9.1% were 33 years or older. In terms of sport experience, 38.5% had 1–5 years, 30.6% had 6–10 years, 21.5% had 11–15 years, and 9.3% had 16 years or more. With respect to athletic level, 79.2% of the participants were amateur athletes, while 20.8% were professional athletes. Additionally, 35.6% of the participants were national athletes, whereas 64.4% had no national-level experience.

### 2.3 Data collection instruments

Within the scope of the study, a demographic information form, the Psychological Wellbeing Scale, the Athlete Attention Scale, and the Competitive Anxiety Inventory were used as data collection instruments. Detailed information regarding these measures is provided below.

Demographic information: This form was used to collect participants’ personal information, including gender, age, athletic level, national team status, and years of sport experience.

Psychological Wellbeing Scale (PWBS): The Psychological Wellbeing Scale developed by Diener et al. (2010) and adapted into Turkish by Telef (2013) was used in this study. The scale consists of eight items rated on a seven-point Likert scale, with total scores ranging from 8 to 56. Higher scores indicate that the individual possesses greater psychological resources and strengths. Exploratory factor analysis revealed that the scale explained 42% of the total variance, with factor loadings ranging between 0.54 and 0.76. Confirmatory factor analysis indicated acceptable model fit indices (RMSEA = 0.08, SRMR = 0.04, GFI = 0.96, NFI = 0.94, RFI = 0.92, CFI = 0.95, and IFI = 0.95). In the original adaptation study, the Cronbach’s alpha coefficient was reported as 0.80 (Telef, 2013). In the present study, the internal consistency of the scale was found to be high, with a Cronbach’s alpha coefficient of 0.93.

Athlete Attention Scale: The Athlete Attention Scale developed by Önal et al. (2024) was used to assess athletes’ ability to direct, sustain, and control attentional focus during competition. The scale consists of 12 items rated on a five-point Likert scale and includes three subdimensions: general arousal, selectivity, and concentration. Total scores range from 12 to 60, with higher scores indicating greater proficiency in attentional processes. According to the exploratory factor analysis results, the three-factor structure explains 64.93% of the total variance, and the factor loadings were found to be significant and strong. In the present study, the internal consistency of the Athlete Attention Scale was high, with a Cronbach’s alpha coefficient of.94.

Competitive State Anxiety Inventory-2 (CSAI-2): The Competitive State Anxiety Inventory-2 developed by Martens et al. (1990) and adapted into Turkish by Koruç (1998) was used to assess athletes’ cognitive and somatic anxiety levels before and during competition. The inventory is structured on a four-point Likert scale and evaluates individuals’ stress levels in competitive settings, performance-related concerns, and physiological responses. In the validity and reliability study conducted by Koruç (1998), the Cronbach’s alpha coefficient of the scale was reported as 0.88. The inventory consists of three subdimensions: cognitive anxiety, somatic anxiety, and self-confidence. In the present study, the internal consistency of the Competitive State Anxiety Inventory-2 was found to be high, with a Cronbach’s alpha coefficient of 0.96.

### 2.4 Data analysis

Data were analyzed using SPSS version 27. In the initial stage of the analysis, the dataset was screened for missing values and outliers. Subsequently, the normality of the variables was assessed using skewness and kurtosis coefficients. Values within the range of ±1.5 were considered indicative of normal distribution (Tabachnick and ve Fidell, 2013).

Pearson correlation analysis was conducted to examine the relationships among the variables. The mediating role of competitive anxiety in the relationship between attentional processes and psychological wellbeing was tested using a regression-based approach with Hayes’ PROCESS Macro. In this context, Model 4 was employed, and the significance of indirect effects was evaluated using a bootstrap resampling procedure with 5,000 samples. The statistical significance of the mediation effect was determined based on the 95% confidence interval; an indirect effect was considered significant if the confidence interval did not include zero (Hayes, 2022).

To assess potential common method bias, Harman’s single-factor test was conducted. The first factor accounted for 32.4% of the total variance, which is below the critical threshold of 50%, indicating that common method bias was not a serious concern in this study. In addition, multicollinearity diagnostics were examined, and variance inflation factor (VIF) values ranged between 1.12 and 1.48, indicating no multicollinearity concerns.

As shown in Table 2, the results of the normality and reliability analyses for the scales used in the study are presented. The skewness and kurtosis values fall within the ±1.5 reference range suggested by Tabachnick and ve Fidell (2013), indicating that the variables meet the assumption of normal distribution. Regarding reliability, the Cronbach’s alpha coefficients were 0.93 for the Psychological Wellbeing Scale, 0.94 for the Athlete Attention Scale, and 0.96 for the Competitive State Anxiety Inventory. These values indicate that all scales demonstrate high internal consistency (Alpar, 2016).

**TABLE 2:** Normality and reliability analysis results.

Variable	*N*	Skewness	Kurtosis	Cronbach’s α
Psychological wellbeing	418	−1.022	1.367	0.93
Athlete attention	418	−0.859	0.789	0.94
Competitive anxiety	418	−0.235	−1.436	0.96

### 2.5 Ethics

This study was approved by the Social and Human Sciences Research Ethics Committee of Aydın Adnan Menderes University (Approval No: E-21315140-050.04-781239; Decision No: 25/01; Date: July 25, 2025).

## 3 Results

In this section of the study, the statistical analyses conducted to test the research hypotheses are presented through tables and figures.

As shown in Table 3, the mean psychological wellbeing score of the participants was 5.83 (SD = 0.89), the mean athlete attention score was 4.25 (SD = 0.60), and the mean competitive anxiety score was 2.05 (SD = 0.64). According to the Pearson correlation analysis, a strong and positive significant relationship was found between psychological wellbeing and athlete attention (*r* = 0.718, *p* ≤ 0.01). In contrast, psychological wellbeing was negatively and significantly associated with competitive anxiety (*r* = −0.574, *p* ≤ 0.01), and athlete attention was also negatively and significantly associated with competitive anxiety (*r* = −0.574, *p* ≤ 0.01). Although the correlations between the variables were statistically significant, the magnitude of these associations remained within acceptable limits and did not indicate potential multicollinearity issues.

**TABLE 3:** Descriptive statistics and correlation analysis results.

Variable	Mean	SD	1	2	3
1. Psychological wellbeing	5.83	0.89	–	–	–
2. Athlete attention	4.25	0.60	0.718**	–	–
3. Competitive anxiety	2.05	0.64	−0.574**	−0.574**	–

**Indicates statistical significance at the 0.01 level (*p* < 0.01).

As shown in Table 4, in the first step of the mediation analysis, the effect of the independent variable (athlete attention) on the dependent variable (psychological wellbeing) was examined. The results indicated that athlete attention had a significant and positive effect on psychological wellbeing (B = 1.06, β = 0.718, *t* = 21.03, *p* ≤ 0.01). The model was found to be statistically significant (F = 442.50, *p* ≤ 0.01), and thus H1 was supported.

**TABLE 4:** Mediation analysis results for the effect of athlete attention on psychological wellbeing via competitive anxiety.

**Outcome variable**	**Predictor**	**B**	**β**	***t***	***P***				
Psychological wellbeing (total effect)	Athlete attention	1.06	0.718	21.03	0.000				
Competitive anxiety	Athlete attention	−0.618	−0.574	−14.29	0.000				
Psychological wellbeing (direct effect)	Athlete attention	0.862	0.579	14.47	0.000				
Psychological wellbeing	Competitive anxiety	−0.335	−0.242	−6.05	0.000				
**Independent variable**	**B**	**SE**	**β**	**t**	***P***	**R**	**R^2^**	**LLCI**	**ULCI**
Dependent variable: competitive anxiety	–	–	–	–	–	–	–	–	–
Athlete attention	−0.618	0.04	−0.574	−14.29	0.000**	0.574	0.329	−0.703	−0.533
Model summary	–	–	–	–	–	–	–	–	–
F = 204.35, *p* = 0.000	–	–	–	–	–	–	–	–	–

Path c, total effect of athlete attention on psychological wellbeing; Path a, effect of athlete attention on competitive anxiety; Path c’, direct effect of athlete attention on psychological wellbeing after controlling for competitive anxiety; Path b, effect of competitive anxiety on psychological wellbeing.

**Indicates statistical significance at the 0.01 level (*p* < 0.01).

In the second step, the effect of the independent variable (athlete attention) on the mediator (competitive anxiety) was tested. The findings revealed that athlete attention had a significant negative effect on competitive anxiety (B = −0.618, β = −0.574, *t* = −14.29, *p* ≤ 0.01). Since the model was statistically significant (F = 204.35, *p* ≤ 0.01), H2 was supported.

In the third step, the mediator variable (competitive anxiety) was included in the model, and the effects of both athlete attention and competitive anxiety on psychological wellbeing were examined simultaneously. The results indicated that the effect of athlete attention on psychological wellbeing remained significant (B = 0.862, β = 0.579, *t* = 14.47, *p* ≤ 0.01), with a reduction observed in the standardized coefficient. In addition, competitive anxiety was found to have a significant negative effect on psychological wellbeing (B = −0.335, β = −0.242, *t* = −6.05, *p* ≤ 0.01). These findings suggest that athlete attention and competitive anxiety jointly exert a significant effect on psychological wellbeing. The model was statistically significant (F = 258.50, *p* ≤ 0.01), and thus H3 was supported.

**FIGURE 2:**
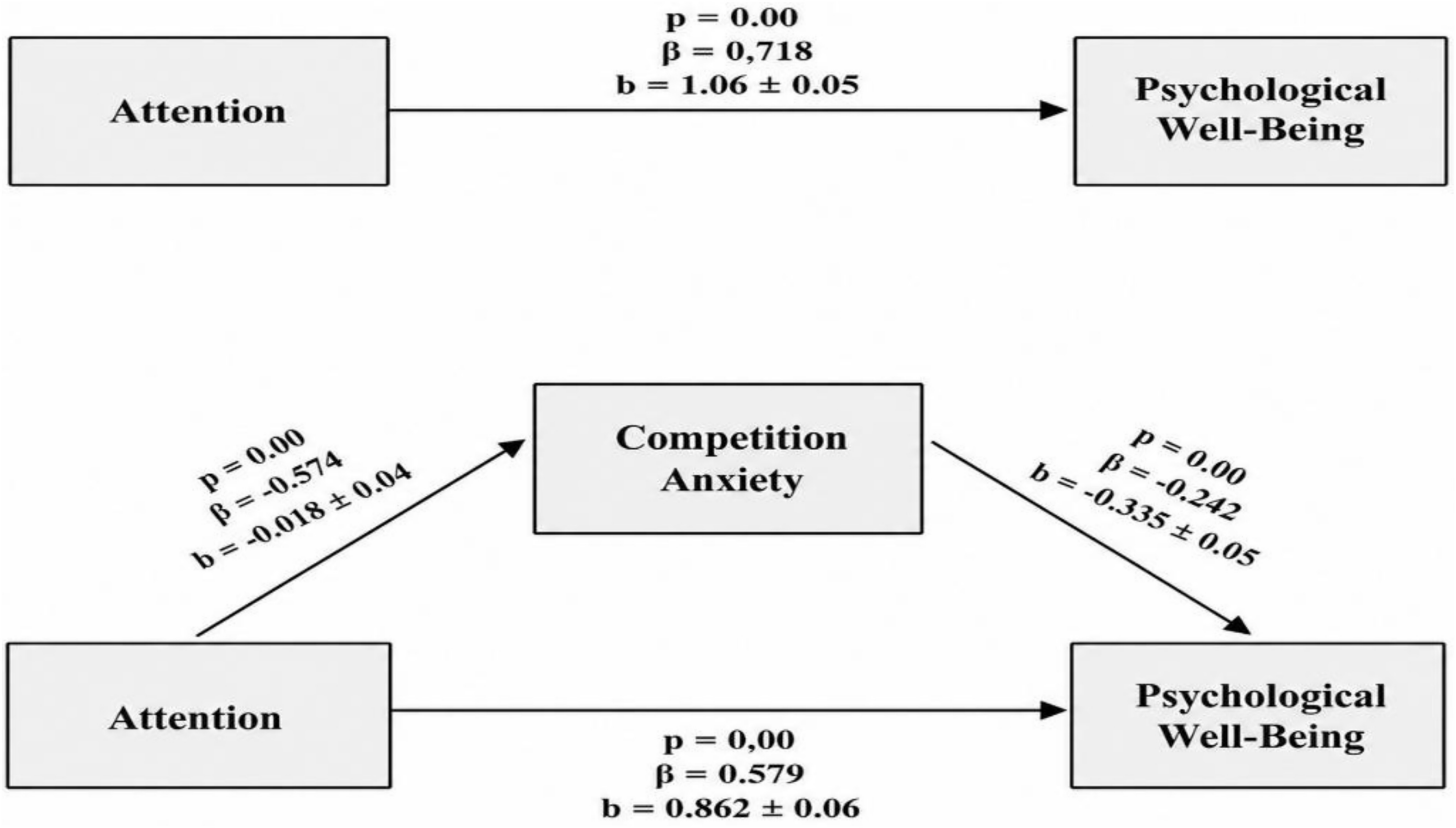
Mediation model of athlete attention, competitive anxiety, and psychological wellbeing.

As shown in Table 5, the bootstrap analysis results indicate that the 95% confidence interval for the indirect effect does not include zero (β = 0.139; 95% CI [0.091, 0.202]). This finding demonstrates that competitive anxiety has a statistically significant mediating effect on the relationship between athlete attention and psychological wellbeing. Furthermore, the direct effect decreases when the mediator is included in the model (from β = 0.718 to β = 0.579), indicating a partial mediation structure. Accordingly, H4 was supported. The final model of the study, along with the effect coefficients, is presented in Figure 2.

**TABLE 5:** Mediation effect of competitive anxiety between variables.

Relationship	Total effect (c)	Direct effect (c’)	Indirect effect (a*b)	BootLLCI	BootULCI	Mediation type
Athlete attention → psychological wellbeing	β = 0.718	β = 0.579	β = 0.139	0.091	0.202	Partial mediation

BootLLCI, bootstrap lower limit confidence interval; BootULCI, bootstrap upper limit confidence interval.

## 4 Discussion and conclusion

This study, which aimed to examine the effect of attentional processes on psychological wellbeing and the mediating role of competitive anxiety in combat sport athletes, was conducted with a total of 418 participants. The findings indicate that combat sport athletes with higher levels of attention exhibit greater psychological wellbeing and lower levels of competitive anxiety. In addition, competitive anxiety was found to play a partial mediating role in the relationship between attention and psychological wellbeing. These results suggest that athletes reporting higher levels of attentional skills also report higher levels of psychological wellbeing and lower levels of competitive anxiety. The observed associations are consistent with a partial mediation model in which competitive anxiety is statistically linked to the relationship between attention and psychological wellbeing. In other words, attention is associated with psychological wellbeing both directly and indirectly through its statistical association with competitive anxiety during high-pressure situations. The relatively strong associations observed among attentional processes, competitive anxiety, and psychological wellbeing are consistent with the theoretical interdependence of these constructs and should be interpreted within this conceptual framework rather than as an indication of methodological inflation.

Overall, the findings suggest that despite high levels of stress and competitive pressure, combat sport athletes with stronger attentional control and higher levels of attentional awareness are able to maintain their psychological adjustment. This supports the notion that attention may exert a regulatory influence on athletes’ mental processes, while anxiety functions as a critical mediating variable within this relationship. In other words, athletes who are able to sustain attention and remain focused on the present moment are better equipped to manage competitive stress, which in turn enhances their overall life satisfaction, happiness, and psychological wellbeing. While no prior study has examined the present set of dependent and independent variables simultaneously within a single model, some studies have investigated these variables in a more limited manner and provide partial support for the current findings. A review of the literature indicates that higher levels of anxiety in athletes are generally associated with increased stress, depression, and burnout, as well as lower levels of wellbeing. Across sport disciplines, including combat sports, anxiety appears to be linked to greater perceived stress and poorer mental health outcomes rather than facilitating psychological adaptation (Quan et al., 2025; Ildefonso et al., 2017; Zhang et al., 2024; Yang et al., 2024; Souter et al., 2018). For example, in high-risk sports (including combat sports), both state and trait anxiety have been shown to significantly increase perceived stress (Quan et al., 2025). Accordingly, the reviewed studies suggest that anxiety does not function as a beneficial mediator for psychological wellbeing; rather, attention-based interventions may help regulate and reduce anxiety levels. In this regard, the findings of the present study provide empirical support for these conclusions. Furthermore, studies examining attention and wellbeing as distinct variables have reported similar patterns of results. Luo et al. (2025), in their study focusing on the mediating role of attention between wellbeing and admiration, found that an attentional bias toward positive stimuli enhances individuals’ wellbeing. In contrast, Blanco and Vázquez (2020), in a study designed in the opposite direction to the present research model, reported that both momentary affect and integrated wellbeing positively mediate attentional orientation toward positive stimuli. Similarly, Xu et al. (2015) concluded that positive attentional bias mediates the relationship between wellbeing and depression by reducing depressive symptoms and enhancing wellbeing. In addition to these findings, a substantial body of literature indicates that various attentional components regulate the relationship between psychological factors and wellbeing. For instance, attentional focusing and a tendency to attend to negative information consistently exert regulatory effects on anxiety and depression, suggesting that poor attentional control and a bias toward negative information increase psychological distress (Li and Fan, 2022; Xu et al., 2015; Mao et al., 2020; Luo et al., 2025). The findings of the present study, consistent with prior research, indicate that combat sport athletes with higher levels of attentional control and awareness are better able to maintain their psychological adjustment. In particular, the mediating role of competitive anxiety in the relationship between attention and psychological wellbeing represents an important contribution that integrates previously fragmented evidence. Research in sport sciences has generally demonstrated a direct association between attention and psychological wellbeing; however, evidence for mediation especially in combat sports remains limited. Some studies emphasize the fundamental importance of attention in supporting psychological skills such as optimal performance, concentration, motivation, and emotion regulation (Martín-Rodríguez et al., 2024; Röthlin et al., 2020; Assadourian et al., 2025). Others report that enhancing attentional control improves psychological wellbeing by increasing resilience and life satisfaction in athletes (Wang et al., 2025; Martín-Rodríguez et al., 2024; Röthlin et al., 2020; Derelioğlu, 2025; Zhong et al., 2025). In addition, attention has been described as interacting bidirectionally with mental health problems, including anxiety, depression, and burnout (Assadourian et al., 2025; Chang et al., 2019). In combat sports, attention and related psychological skills are often considered indispensable for success. However, a recent study reported that attention did not exhibit a significant direct mediating effect on psychological skill level or wellbeing, although higher levels of attention were observed in certain combat sports (e.g., Muay Thai) (Gülsoy and Erhan, 2024). Furthermore, systematic reviews suggest that mental training, including attention-based interventions, yields beneficial psychological outcomes in combat sport athletes; nevertheless, evidence specifically linking attention to wellbeing remains limited, highlighting the need for further research (Andreato et al., 2022).

### 4.1 Conclusion

This study provides evidence that attentional processes are associated with psychological wellbeing in combat sport athletes through both direct and indirect statistical pathways involving competitive anxiety. The findings indicate that attentional processes have a significant positive effect on psychological wellbeing (β = 0.718), while competitive anxiety partially mediates this relationship. The reduction in the direct effect (β = 0.579) and the significance of the indirect effect (β = 0.139; 95% CI [0.091, 0.202]) confirm a partial mediation structure. These results suggest that attentional processes are closely associated with psychological wellbeing and that competitive anxiety may represent an important statistical pathway linking these variables. Overall, the findings highlight the importance of considering attentional control and competitive anxiety together when examining psychological outcomes in combat sport athletes.

### 4.2 Strengths and limitations

A key strength of this study is that it examines attentional processes, competitive anxiety, and psychological wellbeing within a single model in a sample of 418 combat sport athletes, and identifies the mediation mechanism using a bootstrap approach. The findings demonstrate that the relationship between attention and wellbeing operates through partial mediation via competitive anxiety, providing integrative evidence for three variables that have largely been examined separately in the literature. In addition, the high internal consistency of the scales (α = 0.93–0.96) and the fulfillment of normality assumptions support the reliability of the analyses.

However, several limitations should be acknowledged. First, the cross-sectional design limits the interpretation of the mediation findings as causal mechanisms. Second, the restriction of the sample to combat sport athletes and the predominance of amateur-level participants may limit the generalizability of the results to other sports and elite populations. Third, the use of self-report measures may increase the risk of social desirability bias. Additionally, potentially influential variables such as age, years of sport experience, athletic level, and national team status were not controlled as covariates, which may have affected the magnitude of the observed relationships. Finally, as the analyses were based on a single time point, the potential impact of temporal changes in attention and anxiety levels could not be examined. Furthermore, although the CSAI-2 is designed to assess competitive state anxiety, it was not administered immediately before a specific competition. Therefore, the anxiety scores should be interpreted as reflecting perceived competitive anxiety within the broader competitive sport context rather than competition-specific pre-event anxiety.

### 4.3 Theoretical contributions

This study provides empirical evidence that the association between attentional processes and psychological wellbeing may be partially explained by competitive anxiety within a statistical mediation framework. The findings support a partial mediation model, demonstrating that the influence of attentional processes on psychological outcomes is shaped not only directly but also through emotion regulation components. In addition, the study tests the cognitive control–anxiety interaction proposed by Attentional Control Theory within a sport context, showing that this theoretical framework can explain not only performance outcomes but also broader constructs such as psychological wellbeing. From this perspective, the findings indicate that attentional control should be conceptualized not merely as a performance-related variable, but as a regulatory process associated with psychological adjustment and wellbeing. Furthermore, by examining attentional processes, competitive anxiety, and psychological wellbeing within a single integrated model, the study provides a more comprehensive explanation of the mechanisms linking these variables. The results suggest that the effect of attentional processes on psychological wellbeing is better understood as a multi-component structure that is strengthened through changes in anxiety levels, thereby reducing conceptual fragmentation in the literature.

### 4.4 Practical contributions

The findings indicate that the relationship between athlete attention and psychological wellbeing is partially mediated by competitive anxiety. This suggests that, in practice, interventions that simultaneously target attentional development and anxiety regulation may be more effective than approaches focused solely on performance. Accordingly, integrating structured attention training (e.g., sustained attention exercises, attentional shifting tasks) with anxiety regulation techniques (e.g., brief breathing exercises, cognitive reappraisal, pre-competition routines) into training programs may be beneficial. Short pre-competition protocols (e.g., 10–15 min of attentional focus and breathing exercises) may help maintain attentional performance while supporting the regulation of anxiety levels. In addition, it is important for coaches and sport psychologists to tailor interventions based on athletes’ individual anxiety profiles. The partial mediation finding suggests that part of the effectiveness of attention-focused interventions operates through reductions in anxiety levels. Therefore, interventions should not only aim to enhance attentional skills but also address anxiety-related triggers. Such integrated approaches in sport settings may contribute not only to performance-related outcomes but also to the development of training environments that support athletes’ psychological wellbeing in a sustainable manner.

## Data Availability

The raw data supporting the conclusions of this article will be made available by the authors, without undue reservation.
